# Hierarchical, Dense and Dynamic 3D Reconstruction Based on VDB Data Structure for Robotic Manipulation Tasks

**DOI:** 10.3389/frobt.2020.600387

**Published:** 2021-02-18

**Authors:** Carlos M. Mateo, Juan A. Corrales, Youcef Mezouar

**Affiliations:** UMR6602 Institut Pascal (IP), Université Clermont Auvergne, SIGMA Clermont, Clermont-Ferrand, France

**Keywords:** robot manipulation, 3D visual perception, dense reconstruction, robot vision, high performance computing

## Abstract

This paper presents a novel approach to implement hierarchical, dense and dynamic reconstruction of 3D objects based on the VDB (Variational Dynamic B + Trees) data structure for robotic applications. The scene reconstruction is done by the integration of depth-images using the Truncated Signed Distance Field (TSDF). The proposed reconstruction method is based on dynamic trees in order to provide similar reconstruction results to the current state-of-the-art methods (i.e., complete volumes, hashing voxels and hierarchical volumes) in terms of execution time but with a direct multi-level representation that remains real-time. This representation provides two major advantages: it is a hierarchical and unbounded space representation. The proposed method is optimally implemented to be used on a GPU architecture, exploiting the parallelism skills of this hardware. A series of experiments will be presented to prove the performance of this approach in a robot arm platform.

## 1 Introduction

**FIGURE 1:**
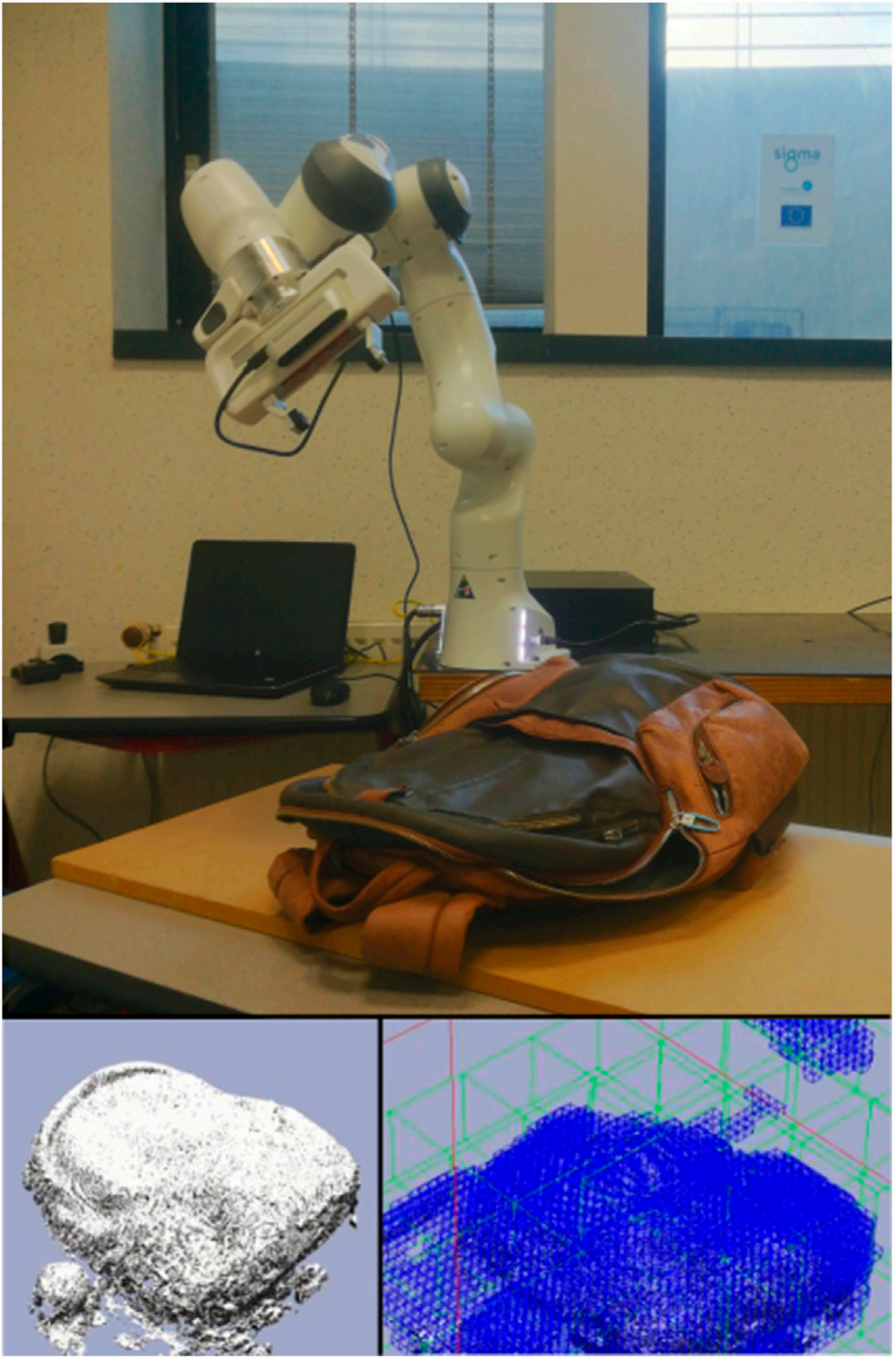
Robot platform used to evaluate our approach. Top: Franka Panda robot equipped with a D435 sensor during the reconstruction of a backpack. Bottom-left: voxelized reconstruction. Bottom-right: topology of the reconstruction.

Industrial robotic research has been extremely prolific in the last decades, with a special interest in applications such as welding, painting and pick-and-place of objects. However, the performance of most of them relies on the precise visual perception of the workplace so that the robot can react in real-time to changes on it. An interesting tool for implementing this perception capability is 3D dense reconstruction. Although 3D dense reconstruction is a well-established field in computer vision and graphics, most of the newly proposed methods are not adapted to the constraints imposed by complex industrial robotic tasks. For instance, when robots need to manipulate deformable objects, current reconstruction methods fail since they are based on the assumption of the presence of rigid objects in static scenarios (Zeng et al. (2013), Whelan et al. (2016) and Puri et al. (2017)). Another well-known problem is drifting in textureless scenarios during the camera pose estimation, which implies erroneous reconstructions. Thus, most of the proposed industrial methods decide to use high-precision and expensive visual sensing setups (Son et al. (2015), Rohrbach et al. (2016) and Zhang et al. (2017)), reducing their applicability in all types of industries. Therefore, we propose to use a new generation consumer depth camera (such as the Intel RealSense D435) installed on the robot so that they can output live half-HD depth maps at high-frequency rates with a low price to implement a precise reconstruction of the objects to be manipulated (Figure 1).

Real-time dense reconstruction presents important challenges when non-delay performance and fine-quality results are required. In particular, the incremental integration of overlapping depth maps into dense volumetric grids is not affordable for sequential methods. This problem has thus been addressed by many works employing different types of data structures accelerated by General Purpose Graphic Processor Units (GPGPU). The most successful methods in the context of hierarchical volumetric grid surface representation are based on Octree data structures, such as the work proposed by Hornung et al. (2013) for robotic collision avoidance tasks. Nevertheless, the main problem with this space representation is its low branching-rate that makes trees considerable deep at low-quality reconstructions. Other approaches more popular in computer graphics are based on N-trees (Chen et al. (2013)) or B-trees (Johnson and Sasha (2002)). Less-known data structures in computer graphics, but one that is quite popular in data science, are the B+ trees. These trees split the topology representation from the stored data (Museth (2013)). The works presented by Hoetzlein (2016) and Wu et al. (2018) are not mere implementations of the VDB (Variational Dynamic B+ trees) data structure for graphics hardware, but they include a major change: data consistency is maintained by using an apron voxels wrap with the neighbor voxels in contrast to use a neighbor index list. In fact, the use of bitmask is not necessary anymore for discovering child nodes.

Implicit volumetric approaches in active sensing have demonstrated fine-quality results, starting with the method by Curless and Levoy (1996), which presents, for the first time, the use of a truncated signed distance field (TSDF). TSDF can also be used at real-time rates (Izadi et al. (2011) and Newcombe et al. (2011)), but a well-known problem of these methods is the lack of memory management. This approach is therefore used just in reduced spaces with modest resolution. To overcome this problem, moving volume variants have been developed (Roth and Marsette (2012)). However, the problem has shifted to streaming out-of-core the data while the sensor moves. A more attractive approach is presented by Nießner et al. (2013), which uses a Hash table to compact the volume grid. However, careful consideration reveals several performance issues according to Museth (2013). Finally, Chen et al. (2013) presents hierarchical data structures that subdivide space more effectively, but they cannot be parallelized efficiently due to their additional computational complexity.

A real-time dense and dynamic 3D reconstruction method implementation, typically used in data science and computer graphics, is proposed to be used in robotics tasks to provide fine-quality results in a hierarchical topology. This new approach has the benefits of dense volumetric grid methods and the multi-level topology representation of hierarchical data structures, but it does not require a memory-constrained voxel grid. This method is based on VDB trees that compress space and allow a real-time integration of new depth images. Additionally, this data structure isolates the implicit surface topology from the data, which is stored densely in cells (called bricks). Although this kind of high-performance hierarchical technique has been proposed for a variety of image rendering, simulations, collision detection tasks (Yang et al. (2017)) and semantic segmentation (Dai et al. (2018) and Hou et al. (2019)), a new extension based on the continuous update of the underlying data is proposed for surface reconstruction in robotics manipulation tasks (Figure 1). All parts of the proposed pipeline (sensor pose acquisition, depth map integration and surface rendering) are performed on GPU hardware, and they are validated by interactive robotic reconstructions of several scenes.

## 2 Terminology of VDB Trees

**FIGURE 2:**
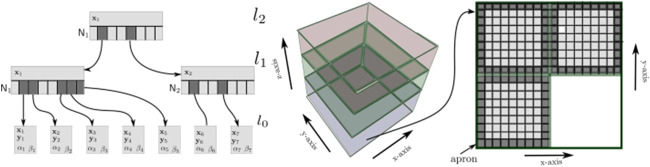
Representation of Variational Dynamics B+ trees adapted to GPU architecture. Left image represents a tree which defines the implicit topology of VDB (for simplification in 2D space), with the following configuration: 2^2^, 2^2^ and 2^2^. Therefore, each node of the internal $l_{1}$ and root $l_{2}$ levels has a child list N of size 16. Nodes in the leaf level $l_{0}$ have an index pointing to the atlas space y in addition to volumetric index x. Atlas is represented at the center of the image as a heap. The right image shows one slice of the atlas space. Apron voxels are used to keep vicinity consistence. Only $pool_{0}$ is shown in this figure.

The proposed method is based on the VDB tree structure to represent a reconstructed scenario in a volumetric grid. VDB exploits spatial coherency of time-varying data to separately encode data values and grid topology (Figure 2). There are no topology restrictions on the sparsity of the volumetric grid and it has a fast random access pattern O(1). In fact, VDB models a virtually infinite 3D index space that allows for cache-coherent and fast data access into sparse volumes of high resolution. The VDB data structure is fundamentally hierarchical, facilitating adaptive grid sampling.

VDB dynamically arranges nodes in a hierarchical data structure, normally a tree (being the grid topology, Figure 2 left), where bricks are leaf nodes at the same fixed depth of an acyclic and connected graph with large but variable branching factors. This makes the tree height-balanced but shallow and wide. This reduces the tree depth and the number of operations to traverse it from the root node to the brick level. The B+ tree is the type used by VDB, which has a variable number of children per node and it can be seen like a traditional B-tree where each leaf contains data keys (index).

The proposed implementation of VDB in Museth (2013) uses a direct access bitmask to guarantee fast and compact direct access to a binary representation of the local topology of a particular node. In contrast, we use the approach presented by Wu et al. (2018), where a pre-reserved and unsorted memory scheme bit masking is not necessary. This unmasked node access provides better computational performance since the resorting of the node list is avoided.

As mentioned before, data values (or voxels) are stored separately from the topology (Figure 2, center and right). The proposed storage scheme presented by Hoetzlein (2016) is used in order to stack the voxels in a 3D heap (atlas), packing them inside bricks. The atlas is allocated in a GPU 3D texture to efficiently access the data. The atlas is resized in the *z*-axis if there is no more empty space in the current atlas. Each brick in the atlas keeps an apron of the nearest neighbor voxels wrapping them. The vicinity consistence in the data layout is thus kept.

Although this scheme of reconstruction is theoretically unbounded in the 3D index space x≡(x,y,z), this is naturally limited to bit-precision and memory constraints. The data encoded in each node consist of (Figure 2): an index x to address the node in a discrete pose inside the volumetric grid V; an index y to map the node with its correspondent brick B in atlas space A; two flags *α* and *β*, which provide information about its activation and visibility; and a list pointing to its children nodes N at the next level. The data value contained inside each voxel $\left\{\bar{d},\bar{w}\right\}\in B$ represents the truncated signed distance field TSDF and the weight. These values are computed by the integration of consecutive depth images D. Since the proposed method is formulated for robotic manipulation, every new D is transformed into the robot base frame by *^b^*M*_c_*.

## 3 Proposed Method

**FIGURE 3:**
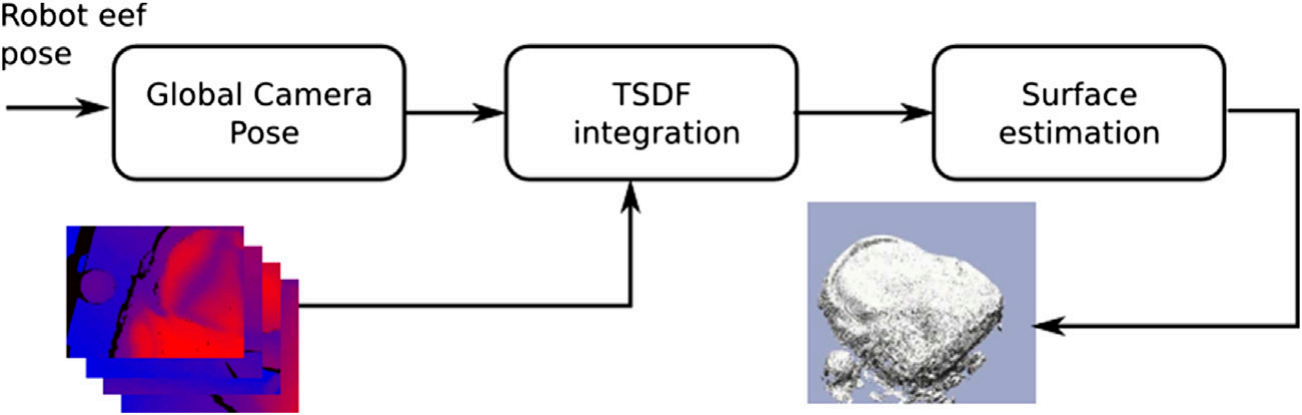
Overview of the proposed 3D dense reconstruction pipeline.

As previously stated, the developed method (Figure 3) is devoted to resolving the reconstruction of dense and dynamic scenarios for robot manipulation tasks. Therefore, a constant and accurate camera pose information retrieval is assumed by the robot direct kinematic solver. This fact makes the method independent of camera pose estimation strategies like in Zeng et al. (2013), Puri et al. (2017), Newcombe et al. (2011), Nießner et al. (2013) and Nguyen et al. (2012). The main reason not to use camera pose estimation is to avoid drifting problems in textureless scenes due to bad error minimization in the Iterative Closest Point (ICP) algorithm (Besl and McKay (1992) and Zhang (1994)).

Therefore, The current global camera pose is therefore obtained by transforming the local camera pose Mec with respect to the current robot end-effector pose: Mbe=Mbe ×Mec. The local pose Mec is estimated using virtual visual servoing (VVS), as in Marchand and Chaumette (2002).

**Algorithm 1:** Topology Manipulation

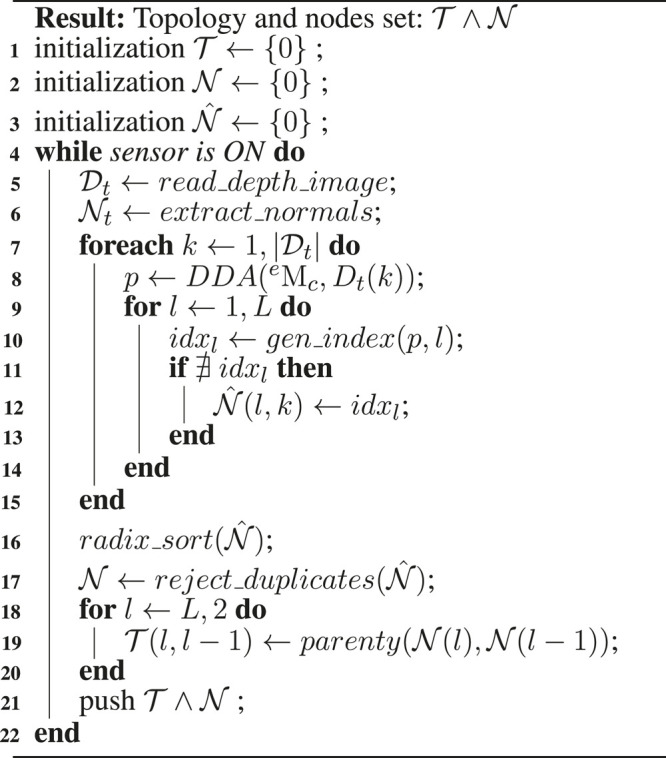

### 3.1 Update of Topology and Atlas Spaces

The volumetric grid topology is updated before the integration of each new depth image. Thereby, new nodes are added to those space quanta that fall inside the footprint of a depth sample z∈D of the truncated region. The *z* are processed in parallel, activating new nodes in the topology and allocating bricks, in atlas space, within the truncation region around the observed surface. Similarly to Nießner et al. (2013), the truncation region is adapted based on the variance of the depth measurements in order to compensate for large uncertainties.

To update the topology, an indexes list of new nodes is created by ray-tracing scanning of V at all tree levels. Note (algorithm 1) that the topology T and nodes set N is an empty structure at the initialization. This scan is also used to update the visibility of those nodes which are already active (α=1) in the topology. Secondly, those nodes belonging to the indexes list created by the ray-tracing are allocated. Thirdly, every new node is linked with its parent in a top-down direction.

**FIGURE 4:**
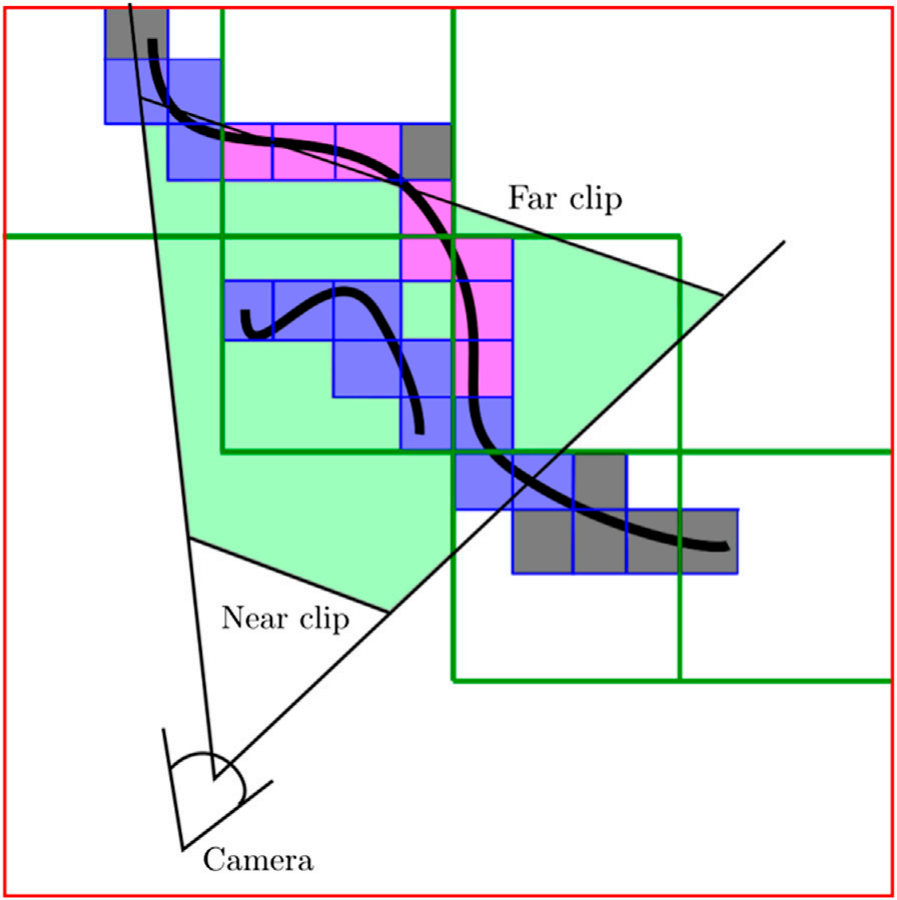
A 2D representation of how nodes are labeled according to the DDA algorithm. In this representation, a hierarchy with $2^{2}$ nodes for levels $l_{1}$ and $l_{2}$ is defined. Leaf nodes are not explored. Camera frustum is defined by near and far clips. Nodes at $l_{0}$ with gray color are outside of the view frustum, purple nodes are active but not visible and the blue ones are visible and active nodes. Neither gray nor purple nodes will be integrated.

A commonly chosen method to implement the ray-tracer is the Digital Differential Analyzer algorithm (DDA, by Amanatides and Woo (1987)) because it interpolates values over an interval between the start and end points. This work defines this interval (i.e the ray bounding region) according to the root node range, in contrast to Nießner et al. (2013) where rays were bounded to the truncation region. This strategy is used to update all visibility information in the current frustum region (Figure 4). The gradient value ∇x used to traverse each ray at level *l* is equal to the resolution at level l−1. This is exemplified in the algorithm 1, from line eight to line 15. This is executed for each instruction in parallel to compute the nodes that hold the depth values $D_{t}\left(k\right)$ measured by the sensor Mec at time *t*. These nodes are computed for each level *l* used to represent the tree.

### 3.2 Depth Image Integration

Depth images are integrated inside of the current volumetric grid: within the bricks whose node position falls inside the camera view frustum and are not occluded (Figure 4). The camera view frustum is defined by the near and far clipping distances. This option keeps a constant computational cost of TSDF integration. Thus, the method performance depends just on the size of the view range and not the density of the reconstruction. In contrast to other works like Nießner et al. (2013), a brick selection strategy is not required since *α* and *β* variables are directly consulted. Thus, all atlas bricks whose node positions are inside the camera range and are visible β=1 are evaluated to implicitly update the volumetric grid.

**Algorithm 2:** Truncated signed distance field integration

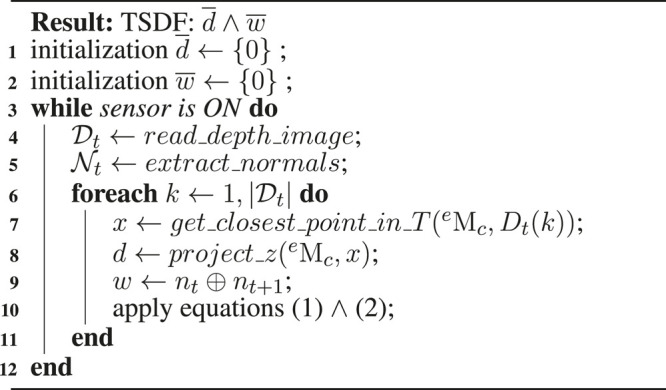

**FIGURE 5:**
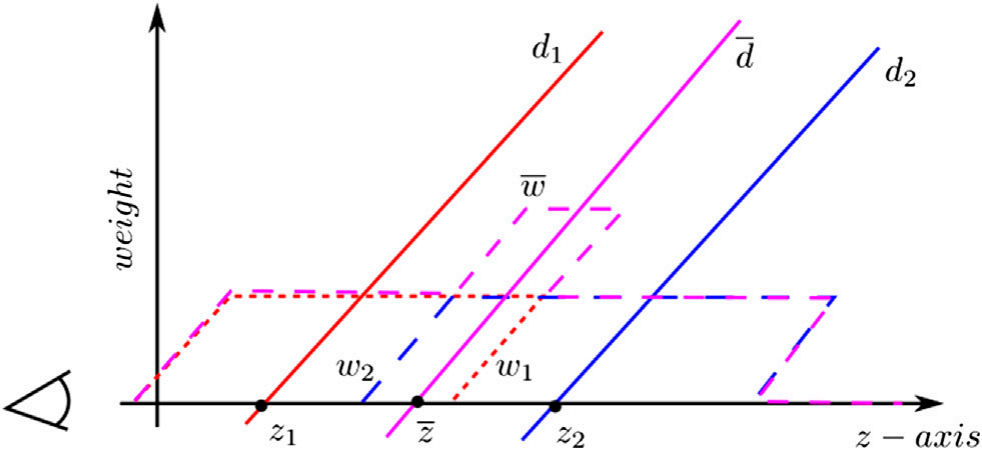
Computation of the TSDF (truncated signed distance field) in one-dimensional space. This figure shows two different measures $z_{1}$ and $z_{2}$ of the same surface spot at different times along *z*-axis in the camera frame. Solid lines show distance fields $d_{1}$ and $d_{2}$ and dash lines represent weights $w_{1}$ and $w_{2}$. Purple lines represent integral distance $\bar{d}$ and weight $\bar{w}$. The surface position $\bar{z}$ is obtained from this integral distance.

Integrating a new depth image involves updating the bricks by re-computing the associated TSDFs and weights (Curless and Levoy (1996)). The calculation of the TSDF is presented in Figure 5 for the special case of 1D. The sensor is positioned at the origin looking down the *z*-axis direction and takes two measurements $z_{1}$ and $z_{2}$ in two different time stamps. The signed distance field $d_{1}\left(x\right)$ and $d_{2}\left(x\right)$ may extend indefinitely in either direction, but the weight functions $w_{1}\left(x\right)$ and $w_{2}\left(x\right)$ bound them behind the range points. Concretely, the weight function *w* shown in (line 9) of algorithm two represents the similarity function based on angular differences between the current normal and the integrated one. ⊕ is thus defined as the dot product of $n_{t}\cdot n_{t+1}$. This implies weighting integration of new depth measurements according to the embedded shape. The weighted combination of the two profiles (Eq. 2) is illustrated in Figure 5 in purple. The integral combination rules are as follows:

$${\bar{d}}_{t+1}\left(x\right)=\frac{{\bar{w}}_{t}\left(x\right){\bar{d}}_{t}\left(x\right)+w_{t+1}\left(x\right)d_{t+1}\left(x\right)}{{\bar{w}}_{t}\left(x\right)+w_{t+1}\left(x\right)},$$ (1)

$${\bar{w}}_{t+1}\left(x\right)={\bar{w}}_{t}\left(x\right)+w_{t+1}\left(x\right),$$ (2)

where, $d_{t}\left(x\right)$ and $w_{t}\left(x\right)$ are the signed distance and weight functions from the *t*th range image. ${\bar{d}}_{t}\left(x\right)$ and ${\bar{d}}_{t}\left(x\right)$ are the cumulative signed distance and weight functions after integrating the *t*th range image.

Note the importance of updating all bricks that fall into the current frustum, irrespective of whether they reside in the current truncation region. This is done to prevent the integration of bricks, which have been added due to surface changes or outliers in the depth map and are no longer observed.

### 3.3 Node Rejection and Surface Generation

This step removes voxel blocks allocated due to noisy outliers and moved surfaces. Node rejection operates on the updated atlas layout to mark a node as rejected and topology layout to remove the nodes. For each brick, a summarization step is performed to obtain both the minimum absolute $\bar{d}$ value and the maximum $\bar{w}$. If the maximum $\bar{w}$ of a brick is zero or the minimum $\bar{d}$ is bigger than a threshold, the associated brick is flagged for deletion. In a second pass, in parallel, all flagged leaves are deleted from the topology. When all deletion operations are successfully done, all nodes in the rest of the tree levels l≠0 are unlinked following a bottom-up pattern. Once both layouts (topology and atlas) have been updated, all nodes are set as non-visible.

Most previous works on dense volumetric reconstruction (such as Nguyen et al. (2012)) extract the implicit iso-surface before rendering the underlying surface. In contrast, the proposed method generates the rendered image of the reconstructed surface directly from the volumetric grid, like in Chen et al. (2013). In order to compute the normal surface, needed for shading, the gradient of the TSDF at the zero-crossing is estimated by using first-order finite differences and trilinear interpolation. The vast majority of samples lie in the same leaf grid due to the use of a shallow tree with relatively large branching factors.

## 4 Results

**FIGURE 6:**
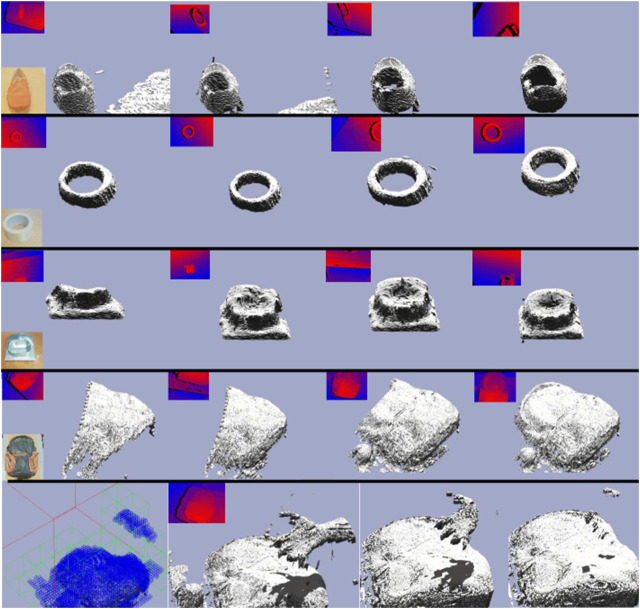
Snapshots of reconstructions experiments for four different objects: a shoe (1st row), tape (2nd row), a small aluminum piece (3rd row) and a backpack (4th and 5th rows).

**FIGURE 7:**
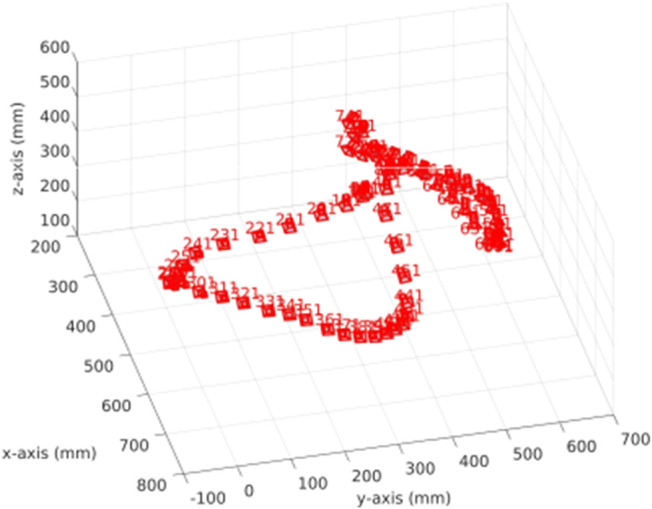
Trajectory of the camera used during the experiments. We sample pose in order to take one of each of the 10 poses.

All the experiments are executed using a laptop PC equipped with an Intel Core i7-6820HQ CPU at 2.70GHz, 32 GB of RAM and an embedded Quadro M2000M GPU. The robot platform is composed of a Robot Franka Panda equipped with an RGBD camera Intel RealSense D435. Four sequences are captured with this set up in order to evaluate the proposed method: a shoe, adhesive tape, a small aluminum piece and a backpack (Figure 6). All the experiments are performed on top of a table situated in z=0 with respect to the robot base. The shoe experiment is the middle size one, 0.3×0.12×0.8 m, of brown leather. The tap experiment is the thin hoop of size 0.1×0.1×0.08 m. The small aluminum piece experiment is used to show how this method can deal with noisy information (measurements corrupted because of the material of the object), the size of the object is 0.07×0.07×0.06 m. The backpack experiment is composed of two objects: an apple and a backpack of 0.47×0.33×0.18 m. The fourth experiment is extended by adding a non-static object (e.g., a human hand) in the scene. The topology configuration for all experiments is the same and it includes for each axis direction: 2^3^ nodes at the root level; 2^3^ nodes at the internal level; and 2^4^ nodes at the leaf level. The voxel resolution is set to $1\;mm^{3}$. The camera pose in all sequences follows the same trajectory (Figure 7) performed by the robot.

Figure 6 presents the reconstruction evolution of all four scenes used in the evaluation. Note that for the surface visualization, the rendering voxels strategy is shown because this representation fits better in reconstructions aimed to measure tasks. Otherwise, the visualization would be misleading. To enrich the voxel representation, internal voxels are also visualized. Since the scene is static with regard to the robot base, the reconstruction is done just in those measures with positive z values.

It is remarkable that the final results in all four scenes have finished without drifting problems. Concretely, in experiment one (shoe), the model evolves from a rough-quality to a fine-quality. The second experiment (tape) is similar to the first one but with an additional difficulty: it is a tiny object, with just a $60\;mm^{2}$ diameter and a 3 mm height. The third experiment (small aluminum piece) is also a tiny object, but it is made of aluminum, which is a reflective material. The last experiment is split into two rows because it presents a more complex scenario. The sequence has two main parts: firstly, the backpack is reconstructed (fourth row) in a first robot trajectory execution (i.e., pass), but later a non-static object (e.g., a human hand) appears in the scenario (fifth row). Even with this occlusion, the previous reconstruction is not affected and it continues to be done successfully in the next robot pass. The shadowing method is used to illustrate occlusion. When the camera is situated for the next pass, the hand goes away from the scene. While the camera does not pass over the region where the hand was, the hand voxels stay. When the camera records once again that scene region, the hand voxels vanish without affecting the backpack reconstruction. More precisely, after the hand is removed from the scene for the first time, the leaf nodes used to code it inside the volumetric grid stay a while. The observed voxels vanish before the nodes because TSDF values inside the voxels become positive, breaking the zero-crossing condition. Afterward, all TSDF reach maximum distance, marking bricks to be rejected.

Unlike other reconstruction methods, this work presents a study of the feasibility of a reconstruction method based on the VDB data structure in robotic tasks (especially manipulation). Because the constraints in this kind of task are mainly knowing the topology of the objects and real-time response, in the following we carry out the following study of computational times. Table 1 presents the time taken by four of the most relevant parts of this method: normal estimation, depth integration, surface generation and the topology update. Time is the average taken for processing a frame. It is interesting to observe that the normal estimation, integration and surface generation are quite constant. This is mainly because these steps are processed in parallel, while normal estimation is computed in image space, the integration and surface generation is computed in atlas space. As a drawback, this method keeps updating the topology in a non-parallel fashion; fortunately, this time tends to decrease linearly as the surface is captured. Once the surface is captured, the time consumption is negligible no matter the motion of the object. This indicates that most of the time is expended when the topology needs to branch.

**TABLE 1:** Algorithm’s profile during experimentation. The table shows the average (per frame) time in milliseconds taken by the most relevant stages during the reconstruction.

	Normal est	Integration	Surface generation	Topology update
Shoe	4.3	15.8	19.5	48.1
Tape	4.1	14.9	18.1	46.0
Alum piece	4.9	13.5	15.4	49.8
Backpack	5.2	18.4	21.6	54.3
Non-static obj	5.7	19.1	23.7	62.8

## 5 Conclusion

A novel dense and dynamic 3D reconstruction method has been implemented based on a hierarchical database structure (GPU oriented) for integrating depth images by truncated signed distance field theory. A qualitative validation of the reconstruction of four different scenes with different properties (materials, size, occlusions, etc.) is performed to show the performance of this method. Current results show that this method provides a stable reconstruction in most situations. But, the method offers a rapid recovery of reconstruction in the case of a failure scenario. Future directions in our research explore the use of this method to simulate material dynamics *in situ*, taking advantage of the GPU-optimized VDB data structure. This will allow us to keep track of non-rigid surfaces while they are being manipulated. Moreover, we will work on the design of active perception using as source data the volumetric grid instead of using directly depth images or point clouds.

## Data Availability

The raw data supporting the conclusions of this article will be made available by the authors, without undue reservation.

## References

[B1] Amanatides J. Woo A. (1987). A fast voxel traversal algorithm for ray tracing. Eurographics. 87, 3–10. 10.2312/egtp.19871000

[B2] Besl P. McKay N. [Dataset] (1992). A method for registration of 3-D shapes. IEEE Tran. Pattern Anal. Machine Intel. 14, 239–256. 10.1109/34.121791

[B3] Chen J. Bautembach D. Izadi S. (2013). Scalable real-time volumetric surface reconstruction. ACM Trans. Graph. 32, 1. 10.1145/2461912.2461940

[B4] Curless B. Levoy M. (1996). “A volumetric method for building complex models from range images,” in Proceeding of 23rd annual conference computer graphics interactation technology–SIGGRAPH ’96. New York, USA, December 4–13, 1996 New York, NY: ACM Press, 303–312.

[B5] Dai A. Ritchie D. Bokeloh M. Reed S. Sturm J. Nießner M. (2018). ScanComplete: large-scale scene completion and semantic segmentation for 3D scans. IEEE Conf. Comput. Vis. Pattern Recognit, 4578–4587. 10.1109/CVPR.2018.00481

[B6] Hoetzlein R. K. (2016). GVDB: raytracing sparse voxel database structures on the GPU. High Perform. Graph. 2016, 109–117. 10.2312/hpg.20161197

[B7] Hornung A. Wurm K. M. Bennewitz M. Stachniss C. Burgard W. (2013). OctoMap: an efficient probabilistic 3D mapping framework based on octrees. Aut. Robots. 34, 189–206. 10.1007/s10514-012-9321-0

[B8] Hou J. Dai A. Nießner M. (2019). “3d-sis: 3d semantic instance segmentation of rgb-d scans,” in Proceedings of the IEEE conference on computer vision and pattern recognition, San Juan, Puerto Rico, June 17–19, 1997 (IEEE), 4421–4430.

[B9] Izadi S. KimHilliges O. Molyneaux D. Newcombe R. Kohli P. (2011). “KinectFusion: real-time 3D reconstruction and interaction using a moving depth camera,” in Proceedings 24th Annual ACM User Interface Software Technology Symposium–UIST, Santa Barbara, CA, USA, October 16–19, 2011 (IEEE) 11, 559–568.

[B10] Johnson T. Sasha D. (2002). The performance of current B-tree algorithms. ACM Trans. Database Syst. 18, 51–101. 10.1145/151284.151286

[B11] Marchand É. Chaumette F. (2002). Virtual visual servoing: a framework for real-time augmented reality. Comput. Graph. Forum. 21, 289–297. 10.1111/1467-8659.t01-1-00588 16805268

[B12] Museth K. (2013). VDB: high-resolution sparse volumes with dynamic topography. ACM Trans. Graph. 32, 1–22. 10.1145/2487228.2487235

[B13] Newcombe R. A. Izadi S. Hilliges O. Molyneaux D. Kim D. Davison A. J. (2011). “KinectFusion: real-time dense surface mapping and tracking,” in 2011 10th IEEE int. Symp. Mix. Augment. Reality, Basel, Switzerland, October 26–29, 2011 (ISMAR 2011 IEEE) 127–136.

[B14] Nguyen C. V. Izadi S. Lovell D. (2012). Modeling kinect sensor noise for improved 3D reconstruction and tracking. Proc.-Model. Process. Vis. Transm. 3DIMPVT. 2012, 524–530. 10.1109/3DIMPVT.2012.84

[B15] Nießner M. Zollhöfer M. Izadi S. Stamminger M. (2013). Real-time 3D reconstruction at scale using voxel hashing. ACM Trans. Graph. 32, 1–11. 10.1145/2508363.2508374

[B16] Puri P. Jia D. Kaess M. (2017). GravityFusion: real-time dense mapping without pose graph using deformation and orientation. IEEE Int. Conf. Intell. Robot. Syst., Vancouver, BC, September 24–28, 2017 (IEEE) 6506–6513.

[B17] Rohrbach A. Rohrbach M. Hu R. Darrell T. Schiele B. (2016). Computer Vision–ECCV 2016. 9905, 1–10. 10.1007/978-3-319-46448-0

[B18] Roth H. Marsette V. (2012). Moving volume KinectFusion. Proc. Br. Mach. Vis. Conf, 112.1–112.11. 10.5244/C.26.112

[B19] Son H. Kim C. Kim C. (2015). 3D reconstruction of as-built industrial instrumentation models from laser-scan data and a 3D CAD database based on prior knowledge. Autom. ConStruct. 49, 193–200. 10.1016/j.autcon.2014.08.007

[B20] Whelan T. Salas-Moreno R. F. Glocker B. Davison A. J. Leutenegger S. (2016). ElasticFusion: real-time dense SLAM and light source estimation. Int. J. Robot Res. 14, 1697–1716. 10.1177/0278364916669237

[B21] Wu K. Truong N. Yuksel C. Hoetzlein R. (2018). Fast fluid simulations with sparse volumes on the GPU. Comput. Graph. Forum. 37, 157–167. 10.1111/cgf.13350

[B22] Yang Z. Gao F. Shen S. (2017). Real-time monocular dense mapping on aerial robots using visual-inertial fusion. Proc. - IEEE Int. Conf. Robot. Autom, Singapore, SI, May 3–June 29, 2017 (IEEE) 4552–4559.

[B23] Zeng M. Zhao F. Zheng J. Liu X. (2013). Octree-based fusion for realtime 3D reconstruction. Graph. Model. 75, 126–136. 10.1016/j.gmod.2012.09.002

[B24] Zhang T. Liu J. Liu S. Tang C. Jin P. (2017). A 3D reconstruction method for pipeline inspection based on multi-vision. Meas. J. Int. Meas. Confed. 98, 35–48. 10.1016/j.measurement.2016.11.004

[B25] Zhang Z. (1994). Iterative point matching for registration of free-form curves and surfaces. Int. J. Comput. Vis. 13, 119–152. 10.1007/BF01427149

